# A burrowing ecosystem engineer positively affects its microbial prey under stressful conditions

**DOI:** 10.1002/ece3.5324

**Published:** 2019-06-13

**Authors:** Laura Bell, Kim Cuddington

**Affiliations:** ^1^ Department of Biology University of Waterloo Waterloo Ontario Canada

**Keywords:** context‐dependent, ecosystem engineering, stress‐gradient hypothesis

## Abstract

1. Species that facilitate others under stressful conditions are often ecosystem engineers: organisms that modify or create physical habitat.
2. However, the net effect of an engineering species on another depends on both the magnitude of the direct interactions (e.g., competition or predation) and the specific environmental context.
3. We used a laboratory system to isolate the trophic and engineering impacts of a predator, the nematode *Caenorhabditis remanei*, on its prey, *Escherichia coli* under different levels of environmental stress. We predicted that under stressful surface conditions the nematodes would positively impact their prey by creating burrows which protected the bacteria.
4. Colony plate counts of *E. coli* indicated that there was a stress‐induced change in the net impact of nematodes on bacteria from neutral to positive. Predator engineering in the form of burrowing allowed larger bacteria populations to survive.
5. We conclude that even in a simple two‐species system a predator can positively impact prey via ecosystem engineering.

## 1 INTRODUCTION

Nontrophic interactions, such as ecosystem engineering, have the potential to alter the relationship between species whose net interactions we have primarily classified according to trophic relationships (e.g., predator–prey). Ecosystem engineering, however, is context‐dependent (Cuddington & Hastings, 2004; Green & Crowe, 2014; Scyphers & Powers, 2013). The modification or creation of physical habitat (Jones, Lawton, & Shachak, 1994; Jones, Lawton, & Shackak, 1997) is sometimes impossible (e.g., a burrower on a rocky shore), while in other situations the alteration of the environment has little positive or negative impact on other species. Therefore, we expect that the sign of the net impact of one species on another may vary depending on their trophic relationship, the ability of the species to alter the physical habitat, and the environmental conditions.

The context‐specific impacts of ecosystem engineering lead to a natural connection to the stress‐gradient hypothesis (Daleo & Iribarne, 2009; Yang, HilleRisLambers, & Ruesink, 2016). The stress‐gradient hypothesis suggests that in low‐stress environments, interactions between plant species tend to be negative, but in high‐stress environments, interactions tend to be positive (Bertness & Callaway, 1994). We follow Brooker and Callaghan (1998), Maestre, Callaway, Valladares, and Lortie (2009), and others in defining the stress referred to as a combination of both stress (factors that limit biomass production) and disturbance (factors that remove biomass).

The facilitating species studied under the stress‐gradient hypothesis are commonly ecosystem engineers. For example, nurse plants shelter seedlings of other species from harsh conditions in the microclimate formed by their canopy, but in the absence of such stress the plants merely compete (Ramírez, Rada, & Llambí, 2015). There has been increased effort to investigate the applicability of the stress‐gradient hypothesis to species other than plants (Bakker, Dobrescu, Straile, & Holmgren, 2013; Dangles, Herrera, & Anthelme, 2013; Fugère et al., 2012). Perhaps the most commonly examined species are sessile animals of the intertidal zone, which may play a similar role as plants in terrestrial systems (Agüera, Koppel, Jansen, Smaal, & Bouma, 2015; Bulleri, Cristaudo, Alestra, & Benedetti‐Cecchi, 2011; Kawai & Tokeshi, 2007). Sessile aquatic animals, like plants, alter the physical conditions through their own physical structure (i.e., autogenic engineering: see Jones et al., 1994). For example, the net effect of tube‐building gastropods (*Vermetus triqueter*) on macroalgae ranged from negative due to competition to positive due to protection from predators with an increasing gradient of consumer pressure (Bulleri et al., 2011).

Other groups of organisms may modify physical conditions through their activities (i.e., allogenic engineering) rather than via their body shape. Only a few authors have addressed the potential application of the stress‐gradient hypothesis to species with this form of environmental modification (Barrio, Hik, Bueno, & Cahill, 2012; Daleo & Iribarne, 2009; Donadi et al., 2013; Travers, Eldridge, Koen, & Soliveres, 2011). The best‐studied mechanism of allogenic engineering is burrowing. Although the stress‐gradient hypothesis normally deals with competitive interactions that switch to facultative interactions, we are unaware of any study that has found this switch for burrowing animals. Some authors, however, have found a switch from neutral to positive interactions. In a meta‐analysis, Barrio et al. (2012) found primarily neutral or positive interactions rather than competition among burrowing herbivores in harsh alpine environments. Donadi et al. (2013) demonstrated a shift from neutral to positive impacts of burrowing cockles on primary producers between moderate and harsher conditions in intertidal flats.

It is possible that switches in the net effects of allogenic engineers may also occur in predator–prey systems. There could be a change from a net negative impact on the prey (predation) to a net positive impact on both species (mutualism) with increasing stress if two conditions are met. First, the predators must have the potential to positively affect prey species under stressful conditions by modifying the physical habitat. Second, the benefits of this positive impact must outweigh the negative effects of consumption. While there are studies which examine the impact of engineering predators on whole community and ecosystem properties (Majdi, Boiché, Traunspurger, & Lecerf, 2014; Sanders & Van Veen, 2011), it is much more difficult to separate out trophic and nontrophic impacts to demonstrate that engineering can act to overcome the negative impact of predation for particular prey species under harsh conditions. There is only one study that supports this hypothesis: in a field experiment, Daleo and Iribarne (2009) found that the net effect of a crab on its marsh grass prey was negative at low stress but positive at high stress. The net positive effect was due to crab burrowing, which improved substrate quality for the plant on high stress, poor substrate sites. The authors described the change in net effect of the crab on the marsh grass as a change from herbivory to facilitation due to increasing stress.

Daleo and Iribarne (2009) wonder whether their finding that an engineering predator can benefit its prey under stressful conditions is isolated to this crab–plant interaction or whether it can be generalized to other systems. Testing this type of hypothesis in the field is difficult, since other species interactions can result in indirect effects that can alter the net impact of the predator. Instead, we examined this question using the nematode, *Caenorhabditis remanei* Sudhaus, and its bacterial prey, *Escherichia coli* Migula in a controlled environment. With only two species in the system, we can be sure that no other species interactions are driving any observed change in interaction direction. Moreover, in this system, we are able to control the presence of stress, and both the trophic and nontrophic interactions between predator and prey.

**Figure 1:**
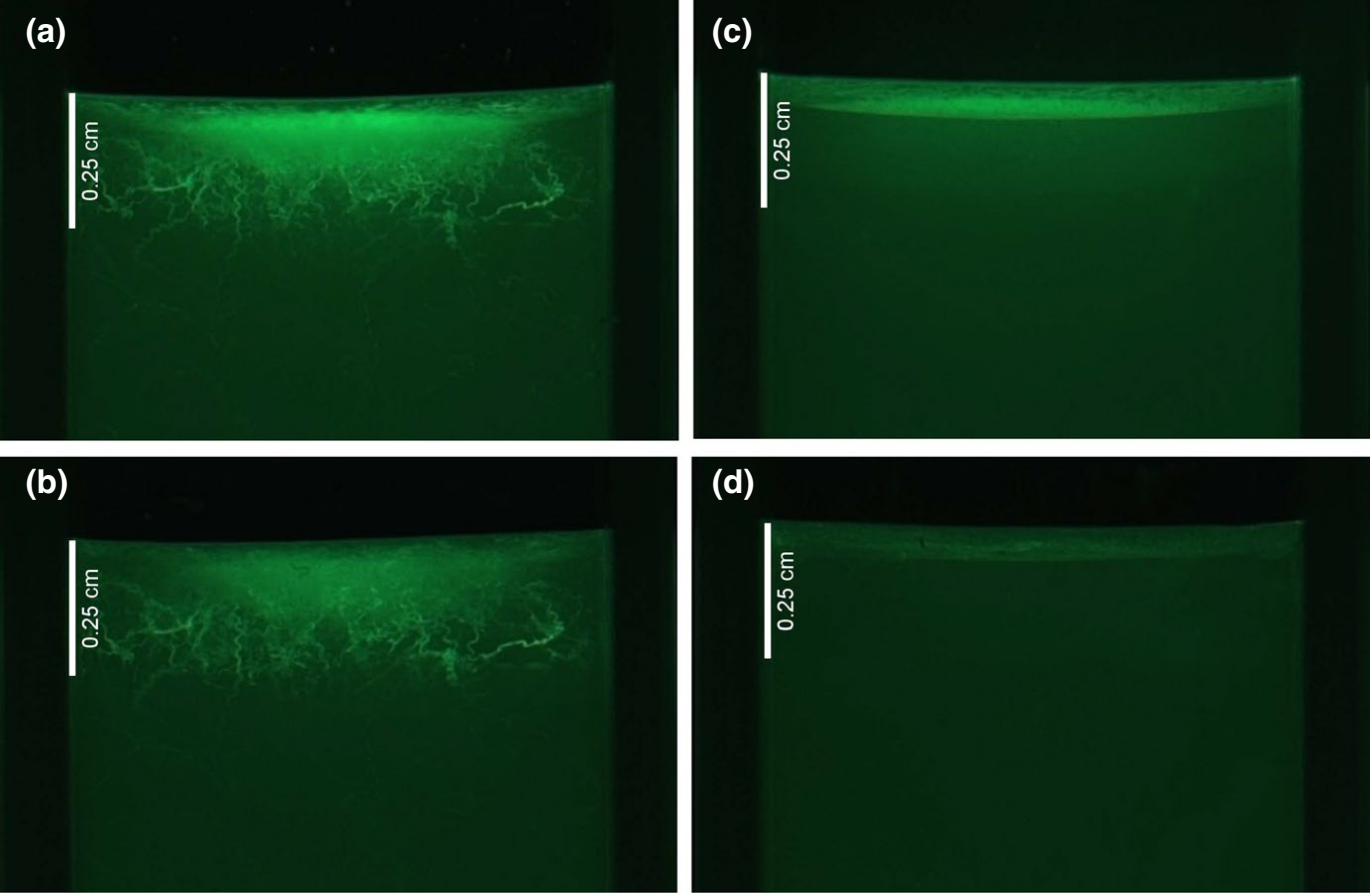
Fluorescent photographs at 4.8 times magnification of 4.5 ml cuvettes filled with agar and inoculated with GFP labeled *Escherichia coli*. Bacteria are visible in burrows made by *Caenorhabditis remanei* nematodes in agar before stress (a) and after stress (b) and are present on only the agar surface in the nonburrowing treatment before stress (c) and after stress (d)

Nematodes can create burrows in an agar substrate (Jensen, 1996 and Figure 1). As the nematodes burrow, it is thought that they spread *E. coli* to the burrows by passing viable cells in their excrement and from their cuticle (Bichai, Barbeau, & Payment, 2009; Chantanao & Jensen, 1969). When an abiotic disturbance is applied to the agar surface, causing both death of some cells and stress for the surviving cells, the nematode burrows could provide refuge. Therefore, the nematode has the potential to negatively impact *E. coli* through consumption and positively impact the bacteria through ecosystem engineering.

We predict that the net effect of *C. remanei* on *E. coli* will be negative under benign environmental conditions or under stressful conditions where the predator cannot burrow. When the predator is able to burrow, its net effect on the prey species will be positive if there are stressful conditions on the agar surface. That is, we predict that the benefits of predator engineering will outweigh any negative effect due to consumption. Therefore, this net pairwise species interaction between an engineering predator and its prey may change from predation (–/+) at low stress to mutualism (+/+) at high stress.

## 2 METHODS

### 2.1 Study species

The predator used in this study, *C. remanei,* is a nematode species with similar biology to the more well‐known hermaphroditic *Caenorhabditis elegans* Maupas. *Caenorhabditis remanei* was selected as a predator because it has obligate sexual reproduction between dioecious individuals, which, by simple segregation of male and female individuals, results in a population of nematodes that will not increase over the duration of the experimental procedure.

Nematodes are cultured on bacterial lawns of *E. coli* (Kiontke & Sudhaus, 2006) grown on an agar substrate. Cultures of nematodes are typically fed *E. coli* OP50, and we used the strain *E. coli* OP50‐GFP (Labrousse, Chauvet, Couillault, Kurz, & Ewbank, 2000). The strain is resistant to ampicillin, which allowed the use of this antibiotic to control bacterial contamination. In addition, OP50‐GFP has been modified to include a green fluorescent protein (GFP) plasmid (pFPV25.1, Valdivia & Falkow, 1996). The green fluorescent protein increased the ease of counting colony‐forming units (CFU) when determining the amount of bacteria present in each treatment. It was also used to indicate *E. coli* presence in nematode burrows.

### 2.2 Stress and engineering experiment

To test the hypothesis that the net effect of an engineering predator on prey can change from negative to positive with increasing stress, the population size of *E. coli* was determined for two levels of stress, with and without nematode predators, and with and without burrowing. Nematodes will engineer burrows if the surface of the agar substrate has been pierced (Stiernagle, 2006). Therefore, treatments included unmodified agar and agar modified by piercing to test the hypothesis that nematode burrowing affects *E. coli*. Since He and Bertness (2014) suggest that the best evidence for the stress‐gradient hypothesis comes from situations with strong, potentially lethal gradients, we simulated stress by briefly applying a square of filter paper to the surface of the agar: both desiccating the surface and removing some bacteria. Therefore, the experiment had a three‐way factorial design for nematode presence or absence, modification of the agar by piercing or unmodified agar as a control, and the application of a filter paper as stress (high stress) or no such application (low stress) for a total of eight treatments (see Table 1). Four trials were completed for a total of 32 replicates per each of the 8 treatments.

**Table 1:** Number of remaining replicates for each of the eight experimental treatments for factors of nematode presence or absence, agar modification (which promotes nematode burrowing), and stress. Each treatment had 32 replicates initially, but replicates contaminated by mold spores, with unexpected burrowing, or with numbers of *Escherichia coli* colony‐forming units that were too small or too large to provide reliable estimates were removed (see Section 2.2)

		Low stress	High stress
Nematodes present	Agar modified	29	28
	Agar control	27	28
Nematodes absent	Agar modified	24	28
	Agar control	23	25

The experimental procedures were time‐consuming, and *E. coli* could increase significantly over this time (Shtonda & Avery, 2006; Virk et al., 2012). Therefore, a randomized block design was used where replicates were processed in one of eight time blocks for a total of 64 per trial (eight replicates per treatment). Treatment replicates were 4.5 ml cuvettes filled with 2.5 ml of 1.0% agar Nematode Growth Medium (recipe similar to Lewis & Fleming, 1995; Stiernagle, 2006) containing the antibiotic ampicillin and the antifungal agent nystatin. The surface dimensions of the agar in the square cuvette were 1.0 cm × 1.0 cm. The cuvettes were inoculated with 10 µl of standardized OP50‐GFP *E. coli* culture with an absorbance of 0.800 ± 0.01 at 600 nm. The *E. coli* was not spread to the edges of the cuvette to discourage nematodes from climbing the sides and to prevent *E. coli* from seeping down between the agar and the cuvette wall. Between steps of the experimental procedure, tightly sealed plastic caps prevented contact with airborne contaminants. Cuvettes were stored in a 20°C incubator.

Twenty‐four hours after adding the *E. coli*, each cuvette was photographed twice, once on each clear side of the cuvette, using a microscope with a UV light (x‐cite Q 120, Lumen Dynamics), a filter for the green fluorescent protein (470 nm), and a camera attachment (AxioCam MRc). Cuvettes with bubbles in the agar, *E. coli* spread out over the entire agar surface, or punctures in the agar were removed from the experiment. For treatments with nematodes, twenty‐four hours after adding the *E. coli*, seven male nematodes were added to the surface of the agar. For treatments without nematodes, the sterilized platinum wire spatula used to transfer nematodes was instead used to mimic the disturbance of the bacterial lawn due to nematode transfer. For modified agar treatments, the center of the agar was pierced to a depth of 1 mm once prior to adding the nematodes.

Nematodes were left to burrow and interact with the bacteria for 96 hr at 20°C. After this period, a second set of photographs was taken to determine if there was burrowing by nematodes in the agar control treatments replicates, and if so, these cuvettes were removed from the experiment. After the 96 hr interaction period, a sterilized square filter paper (1 cm by 1 cm) was pressed onto the agar surface of the cuvette and then removed after 30 min for high‐stress treatments. All replicates were briefly uncapped even if they were not in the high‐stress treatment. The stress treatment may also have removed nematodes from the cuvette. To determine how many nematodes remained in the cuvettes following the surface disturbance, they were counted under a dissecting microscope. After the application of the filter paper, the cuvettes were returned to the incubator for a 24‐hr recovery period.

The colony plate count method was used to estimate the amount of bacteria in each cuvette after the 24‐hr recovery period. The agar in the cuvettes was homogenized using 1.0 ml of sterile saline solution, glass beads, and a vortex mixer. A dilution series was performed for an aliquot from each cuvette, and bacteria were spread onto Petri plates containing Luria agar (Bertani, 1952; Gerhardt, Murray, Wood, & Krieg, 1994). The plates were incubated at 30°C for 23 hr, and then the Petri plates were photographed using a fluorescent filter to detect the OP50‐GFP bacteria. The number of colony‐forming units per plate was counted using a cell counter plug‐in for the computer program ImageJ (Rasband, 2016; De Vos, 2010).

Before analysis, replicates that had one or more contaminant spores visible in the cuvette agar were removed. In addition, replicates where the number of colony‐forming units on all counted Petri plates for all dilution levels was outside of the range where reasonable density estimates could be obtained were also removed (i.e., between 30 and 300 CFUs, Goldman & Green, 2015). Finally, one‐time block of the first trial was also removed because the mean number of colony‐forming units per ml for this block was more than two standard deviations less than the mean for all blocks. This difference was likely due to human error when creating the dilution series. As a result, the number of remaining replicates per treatment ranged from 23 to 29 (Table 1).

### 2.3 Data analysis

The data were analyzed using a generalized linear model in the statistical package R (R Core Team, 2013). After CFU data were transformed using the natural logarithm to normalize model residuals, a generalized linear model, with factors of stress, nematode presence and agar manipulation, where the data were blocked by time trial, was used to determine if nematodes had a positive impact on the density of their bacterial prey (CFU/ml) in the presence of stress. A generalized linear model with factors of stress and agar manipulation was used to test for differences in the number of nematodes in each treatment. Post‐test comparisons of means from different treatments were completed using the Tukey HSD test.

## 3 RESULTS

**Figure 2:**
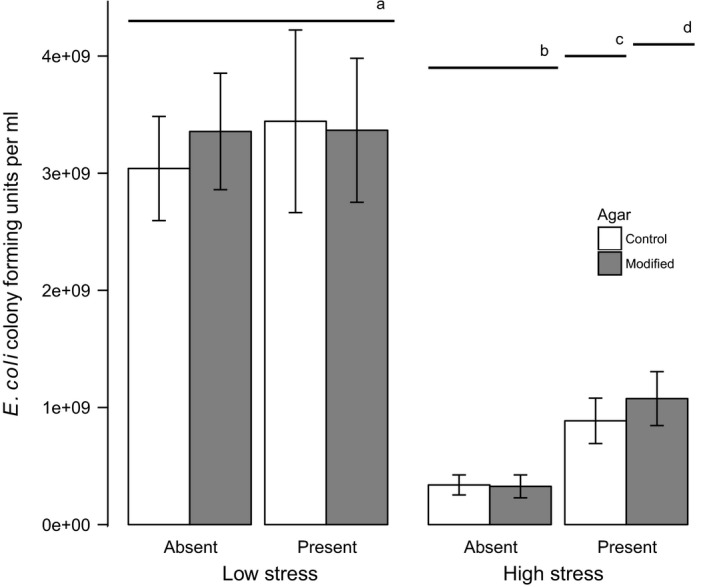
Mean number of *Escherichia coli* colony‐forming units per ml of agar with error bars showing ± one standard deviation. Modified agar that allows ecosystem engineering is represented by shaded bars. The labels “absent” and “present” indicate if nematodes were added, while “low stress” and “high stress” indicate if stress was applied in the form of a physical disturbance to the agar surface. Treatments with shared letters do not differ significantly from each other as indicated by a Tukey's comparisons of means

There were significant and interacting impacts of nematodes, engineering and stress on *E. coli* as measured by the number of colony‐forming units (CFUs) per ml of agar in the cuvettes (*F*_10, 201_ = 10.3, *p* < 0.05, Figure 2).

Stress reduced the number of CFUs of bacteria per ml of agar for all treatments. When there were no nematodes and no modification of the agar, the high‐stress treatments had a significantly lower number of *E. coli* CFUs measured 24 hr later (Tukey HSD, mean = 3.2×10^9^ and 3.2×10^8^ *E. coli* CFUs for low stress and high stress, respectively, Figure 2). Experimental manipulation of the agar did not affect these results. There was no difference between treatments with and without agar modification in high‐stress conditions where nematodes were absent (mean = 3.3 × 10^8^ and 3.1 × 10^8^ *E. coli* CFUs for control agar and modified agar, respectively).

There was a positive impact of nematodes on bacteria in high‐stress treatments (Figure 2), and bacteria were observed in nematode burrows following the application of disturbance (Figure 1). A smaller net positive effect under stress was found when nematodes were unable to burrow; however, the number of colony‐forming units was significantly lower in this case (mean = 8.7 × 10^8^ and 1.0 × 10^9^ *E. coli* CFUs for control agar and modified agar, respectively, Figure 2).

Since there was no net effect of nematodes on bacteria at low stress (Tukey's comparison of means and Figure 2), we conclude that burrowing did not increase the equilibrium density of the bacteria in the cuvette environments. However, the number of nematodes observed after the application of stress differed significantly among treatments (*F*_4, 53_ = 3.9, *p* = 0.01) when data were blocked by trial. The number of nematodes remaining on control agar was higher than on modified agar by approximately one nematode (Tukey's comparison: mean = 2.9 and 2.1 nematodes for control agar and modified agar, respectively). This finding could affect the interpretation of results, but seemed at odds with our expectation that nematodes might be more sheltered in treatments where they could burrow. Subsequent counts with known numbers of animals found that there were detection difficulties in treatments with burrows. As a result, we suspect that there was in fact no significant difference in nematode number between treatments.

## 4 DISCUSSION

We demonstrate for the first time that predators can positively impact their prey through engineering in the absence of other species interactions. However, we also show that the magnitude of this benefit depends on environmental conditions. The positive impact of the nematode *C. remanei* on its microbial prey, *E. coli* was greatest when the nematodes were able to burrow and surface conditions where stressful, supporting the extension of the stress‐gradient hypothesis to systems involving both allogenic ecosystem engineers and microbial organisms. However, the beneficial effect of this ecosystem engineering disappeared when environmental conditions were benign. Therefore, in natural systems, we may also expect context‐dependent net interaction rates. In particular, our intuition regarding the net effect of a predator on a prey may be mistaken, especially when extreme conditions such as drought, excessive wave action, or heat waves occur.

### 4.1 Nematode engineering impacts on *E. coli*

Unlike previous work, our laboratory study allowed us to manipulate a two‐species system so that we could tease apart the trophic and engineering links that contribute to the total interaction rate under different environmental conditions. When we manipulated both the ability of our engineer species to burrow and the stress levels in the environment, the net impact of the predator on its prey became positive under stressed conditions. The positive impact of nematodes was largest when nematodes created burrows, which contained a visible *E. coli* population under magnification (Figure 1). We therefore conclude that these predator burrows provided refugia for bacteria from the stressful conditions on the surface of the agar.

We predicted that nonengineering predators would have a negative impact on their prey under stressful conditions. Unexpectedly, all high‐stress treatments with predators had significantly higher bacterial populations than control treatments without predators. Therefore, nematodes in the nonengineering treatment may have created other refugia for *E. coli*. For example, the bodies of the nematodes may have shielded bacteria from the surface disturbance by covering cells underneath them, nematodes may have harbored viable cells in their gut tract (Bichai et al., 2009; Chantanao & Jensen, 1969), or the shallow grooves that nematodes created on the surface of the agar may have been sufficient to protect the *E. coli* from either removal or desiccation by the filter paper.

In answer to the question posed by Daleo and Iribarne (2009) of whether an engineering predator could benefit its prey in systems other than the crab–marsh grass system they examined, we provide a conditional yes. Moreover, we demonstrate this positive impact in a system where we can separate trophic and engineering effects, and where no other species interactions could have influenced the results. However, we did not see the dramatic stress‐induced flip from a net negative interaction to a net positive predation that we had expected. Instead, the net impact of nematodes on *E. coli* changed from neutral to positive in the presence of stress.

Surprisingly, the predator had a neutral impact on its prey under low‐stress conditions. There is no question that *C. remanei* feeds on *E. coli* (Avery & Shtonda, 2003; Avery & You, 2012; Bichai et al., 2009; Chantanao & Jensen, 1969; Stiernagle, 1999). In fact, these bacteria are normally the only food source provided to laboratory populations of nematodes, while all others are suppressed via the use of antibiotics. Our experiment even required a restricted number of predators so that nematodes would be less likely to consume all bacterial cells in the small cuvette environments. Moreover, we removed all replicates with any evidence of contamination. It is possible that the nonlinear relationship between the absorbance of the inoculated *E. coli* and the measured number of colony‐forming units following homogenization of the agar prevented the detection of small differences in response at very high bacterial density (see Appendix S1). In addition, nematodes can excrete viable bacteria cells (Bichai et al., 2009; Chantanao & Jensen, 1969), and food‐saturated nematodes may produce a particularly high number of viable cells in their excrement (Chantanao & Jensen, 1969) thus reducing the negative impact of predation.

### 4.2 Engineering in microbial systems

Uniquely, we find that there is a significant interaction between ecosystem engineering and stress for microbial populations. Therefore, we suggest that the stress‐gradient hypothesis may be quite broadly applicable across species groups. However, we do note that generalization from this study to microbial systems should be done with caution. The stress‐gradient hypothesis was formulated to describe the impact of stress on the overall trend among many species in a given community. Like other authors, we have applied it here to a specific pairwise species interaction, but the specific details of a single interaction may determine the outcome. Under a different form of stress, one that was not mitigated by burrowing, we would not expect benefits from the presence of these predators. It is even conceivable that burrowing could amplify the negative impacts of other forms of stress. There is work to suggest that burrowing may have both positive and negative effects on microbial populations. Burrowing ecosystem engineers alter water fluxes and chemical gradients that subsequently impact microbial processes in sediments and soils, and so could both increase or decrease microbial populations (Gutiérrez & Jones, 2006; Mermillod‐Blondin & Rosenberg, 2006). Such effects of engineering could either amplify or mitigate different forms of stress.

Expanding the study of the stress‐gradient hypothesis to microbes was suggested by He and Bertness (2014) to increase our understanding of the mechanisms governing species interactions. We further suggest that using microbial systems will also aid in our understanding of ecosystem engineering. There have been only a few efforts in this direction (Bowker, Soliveres, & Maestre, 2010; Navel, Mermillod‐Blondin, Montuelle, Chauvet, & Marmonier, 2012). In addition, microbial studies are often compatible with a laboratory system that offers some practical advantages. Disentangling the effects of trophic and engineering links is quite difficult in the field (Majdi et al., 2014; Sanders & Van Veen, 2011). For example, Daleo and Iribarne (2009) were unable to exclude other predators from experimental plots. In addition, stress levels can vary uncontrollably in field conditions. While ecologists are sometimes dismissive of model system studies, using species that can be cultured in the laboratory may help refine our understanding of the mechanisms involved in stress and engineering‐induced change in net species interactions.

### 4.3 Broader implications for variable effects of engineering and trophic links

Like other authors, we find that the stress‐gradient hypothesis may be applied to allogenic ecosystem engineers. A change in pairwise species interactions, whether from negative to positive or neutral to positive, over stress gradients may have important implications for prediction and management. Crain and Bertness (2006) propose that species may be facilitated by ecosystem engineers so that they may persist under stressful environmental conditions that they would otherwise not survive. Our work reinforces previous studies that report that burrowing animals can benefit other species (Machicote, Branch, & Villarreal, 2004; Meysman, Middelburg, & Heip, 2006; Pike & Mitchell, 2013) but similar to previous work in natural systems (Barrio et al., 2012; Daleo & Iribarne, 2009; Donadi et al., 2013), we only find beneficial impacts of burrowing in stressed environments.

Our work examining both trophic and engineering effects of predators suggests this conclusion may be extended in a further, perhaps more unexpected, direction. Predators may provide a net benefit to prey under stressful conditions. For example, in areas subject to drought, the endangered Hine's emerald dragonfly may have an obligate mutualistic relationship with a predatory crayfish (Pintor & Soluk, 2006). The crayfish burrows provide an aquatic environment for dragonfly larvae in dry conditions. A traditional management plan for an endangered species might remove the predatory crayfish, but, depending on the frequency of drought, this action would probably have negative impacts on the survival of the dragonfly population. Therefore, stress‐induced changes in the net effect of predator‐prey relationships may play a critical role in the preservation of rare and endangered species.

In this simple system, we find strong evidence that engineering predators can benefit their prey by altering the physical environment. However, we also find evidence of a benefit of nonburrowing predators under stress. If the interaction of trophic and nontrophic effects, as well as stress‐induced changes in these links, are common across most species groups, and our work adds to the weight of evidence that they are, then it is no longer sensible to rely heavily on categorizations such as predator and prey. We conclude that it is important to distinguish between the potentially positive or negative components of a pairwise species interaction, and the net effect of one species on another. We suggest that ecologists embrace both the diversity of links between species and the context‐dependence of net interaction rates.

## CONFLICT OF INTEREST

None declared.

## AUTHOR CONTRIBUTIONS

KC and LB conceived and designed the experiments. LB performed the experiments and lead the writing of the manuscript. KC and LB analyzed the data. Both authors contributed critically to the drafts and gave final approval for publication.

## Data Availability

Data available from the Dryad Digital Repository: https://doi.org/10.5061/dryad.tt6v3jg.
