# Supplementary material for: A burrowing ecosystem engineer positively affects its microbial prey under stressful conditions
Source: Ecol Evol. 2019 Jun 13;9(13):7704–11. doi: 10.1002/ece3.5324 (PMC6635926; doi:10.1002/ece3.5324)

Supporting Information – Bell & Cuddington

We conducted an experiment to determine if the agar homogenization procedure produces *E. coli* colony plate counts that accurately quantify bacterial abundance. An aliquot of 1 ml of five different concentrations of *E. coli* OP50-GFP was added to spectrophotometry cuvettes. The concentrations were 0.00, 0.01, 0.1, 0.4 and 0.8 absorbance at 600 nm, standardized to a blank of sterile broth. There were three replicates per concentration that were divided by three time blocks with one replicate of each concentration per block. Following this, 1.5 ml of 1.67% agar was added to the cuvettes to make a final agar concentration of 1.0% and a final volume of 2.5 ml to be consistent with the experimental design described in the main text. An hour after cuvettes were made, the homogenization procedure described in the main text was used to determine the number of colony forming units for each cuvette. In this case, the count plates were made using the pour plate technique rather than the spread plate technique (bacteria cells grow within the agar rather than on the agar surface). Other differences from the main text method include: 1.2 ml of saline was added to the cuvette rather than 1.0 ml, and a 50 ul initial aliquot was taken from the sample rather than 100 ul.

After incubation, photographs of two quarters of the Petri plate were taken and the fluorescent colony forming units (CFUs) were counted using software program ImageJ. The number of colony forming units per ml was calculated using a standard of 2.5 g of agar for each cuvette.

To determine if the *E. coli* estimates from the colony plate counts were predicted by the concentration of *E. coli* initially inoculated, we conducted a linear regression with the data blocked by time and checked for normality of model residuals. Since there were no significant impacts of the time block of the experimental replicates, subsequent analyses were completed with grouped data. A linear regression with a zero intercept (CFU=a*Absorbance) indicated that the slope was significantly different than zero (Fig_1_SuppInfo: F3,11 = 18.6, $p < 0.05$, adjusted R2 = 0.8). However, an examination of the residuals suggested that a non-linear, saturating response might be a better fit to the data. Accordingly we fit the model CFU=b*Absorbance/(c+Absorbance), which proved to be the preferred description of the data (AIC linear model = 592 AIC nonlinear model = 576). All analysis were completed in R (R Core Team 2013) using the nonlinear least squares function (nls).

Figure 1 Supporting Information: Nonlinear (blue solid line) and linear regression (red dashed line) of the estimated number of *E. coli* colony forming units per ml of cuvette agar by absorbance (at 600 nm) of *E. coli* culture initially inoculated (open circles). Colony forming units were estimated using colony plate counts that diluted bacteria following a homogenization procedure similar to that used in the experimental design described in the main text.

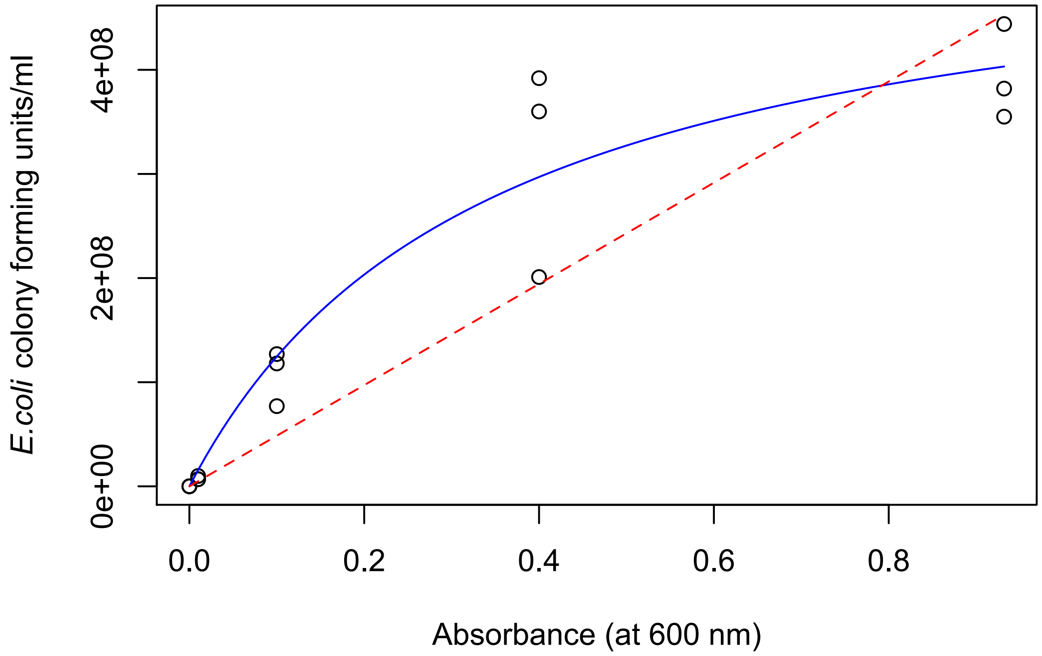

Supplement: Supplementary file 1 [file ECE3-9-7704-s001.pdf]
